# Human and Marine Host Defense Peptides for Healthy Skin

**DOI:** 10.3390/md24040134

**Published:** 2026-04-10

**Authors:** Svetlana V. Guryanova, Oksana Yu. Belogurova-Ovchinnikova, Tatiana V. Ovchinnikova

**Affiliations:** 1 M.M. Shemyakin and Yu.A. Ovchinnikov Institute of Bioorganic Chemistry, Russian Academy of Sciences, 117997 Moscow, Russia; svgur@ibch.ru; 2 Medical Institute, RUDN University, 117198 Moscow, Russia; 3 Moscow Center for Advanced Studies, 123592 Moscow, Russia; belogurova-ovchinnikova.oyu@mipt.ru; 4 Department of Biotechnology, I.M. Sechenov First Moscow State Medical University, 119991 Moscow, Russia

**Keywords:** antimicrobial peptides, host defense peptides, antibacterial, antibiofilm, antifungal, immunomodulatory, antioxidant, antiaging, antiphotoaging, wound healing

## Abstract

The skin serves as the first line barrier of innate immunity, protecting the body from external influences and maintaining its homeostasis. Exogenous and endogenous stress factors alter the structure and functional properties of the skin. The search for compounds capable of counteracting these processes has allowed the identification of peptides as promising ingredients of products for medicinal and cosmetic applications. This review comprehensively examines the mechanisms of action and dermatological applications of two distinct classes of natural products—endogenous human peptides and those derived from marine organisms. Human peptides exhibit numerous biological functions, including antimicrobial and immunomodulatory ones, as well as promoting antioxidant protection and wound healing. Microbiome-associated peptides are an underestimated but powerful regulator of skin aging through immunomodulation, inflammation control, barrier function maintenance, and selection of the proper microbial community. Peptides from marine organisms exhibit significant structural diversity and a broad spectrum of biological activity, including regenerative effects and effects on antibiotic-resistant microorganisms. This review summarizes current data obtained from in vitro, ex vivo, and clinical studies demonstrating a broad potential of peptides for maintaining skin health. Both peptide classes represent powerful, targeted strategies for innovative dermatological interventions aimed at promoting skin rejuvenation, protection, and overall homeostasis.

## 1. Introduction

The skin provides the first line of body defense system, employing both physical and chemical barriers to repel pathogens. In the human epidermis, keratinocytes play a key role in epidermal repair and maintaining skin integrity [1]. They secrete cytokines and chemokines, promoting the recruitment of macrophages and polymorphs to the skin injury site, and express Toll-like receptors (TLRs), recognizing pathogen-associated molecular patterns (PAMPs). Different signaling molecules, including interleukins IL-1β and IL-6, tumor necrosis factor (TNF), are involved in the immune response intermediated by keratinocytes [2]. Further understanding of molecular mechanisms involved in cutaneous immune responses is needed to develop novel therapeutic agents for the treatment of skin diseases [3].

Among other biological functions, host defense peptides, also known as antimicrobial peptides (AMPs), play a pivotal role in cutaneous immunity [4,5,6]. Skin AMPs are short, often cationic peptides produced by keratinocytes, sebocytes, sweat glands, immune cells, and other skin components. They exhibit broad-spectrum activities against bacteria, viruses, and fungi [7,8,9,10]. These compounds form a part of the skin’s chemical shield alongside other factors like low pH, helping to maintain a healthy microbiome and prevent infection.

Marine organisms, which inhabit microbe-rich and extreme environments, produce a diverse array of potent AMPs [11,12]. These marine peptides frequently display a broad antimicrobial spectrum and unique mechanisms of action [13,14,15,16,17]. Notably, some marine AMPs remain active in high-salt or harsh conditions that typically inactivate human AMPs. Given the increasing prevalence of antibiotic resistance, marine host defense peptides are being investigated as next-generation therapeutics for skin infections, wound healing, and anti-aging skincare. These peptides can eliminate drug-resistant microbes by targeting membranes, a mechanism to which bacteria have difficulty developing resistance, and can also modulate inflammation and promote tissue repair. This review examines the role of major human host defense peptides in skin health and disease, discusses potential applications of marine-derived peptides for skin disease prevention and mitigation of skin aging, addresses their possible interactions in the human body, and finally considers the input of the skin microbiome and microbial peptides in skin homeostasis and aging.

## 2. The Role of Human Host Defense Peptides in Skin Health

Human skin produces a battery of host defense peptides. These include dermcidin, defensins, cathelicidin (LL-37), and some peptides derived from proteins, for instance, from lactoferrin. Together, they provide antimicrobial protection and regulate inflammation on the surface of the skin [18]. In healthy skin, many of these peptides are constitutively expressed at low levels and can be rapidly upregulated in response to injury or infection. They create an antimicrobial layer on the surface that selectively curtails pathogens while generally co-existing with the commensal flora [19]. An overview of selected human skin AMPs and their roles is followed by a discussion of strategies to maintain a healthy balance of human endogenous and exogenous peptides.

### 2.1. Dermcidin

Dermcidin is a unique antimicrobial peptide constitutively secreted by human eccrine sweat glands into the sweat and transported to the skin surface. It was originally discovered in 2001 as a novel peptide active against a variety of pathogenic microorganisms [20]. Dermcidin is produced as a precursor (pre-DCD) containing 110 amino acid residues and then proteolytically processed in sweat into one or more active peptides about 47 amino acid residues in length. These processed dermcidin peptides are remarkable for their ability to function under harsh conditions of human sweat: they remain active across a broad pH range and at high salt concentrations, similar to sweat composition. This allows dermcidin to exert antimicrobial effects even as sweat dilutes and evaporates on the skin surface [21,22,23].

In terms of activity, dermcidin-derived peptides have a broad-spectrum efficacy [24]. They can kill both Gram-positive and Gram-negative bacteria, as well as some fungi, by mechanisms that do not necessarily involve conventional membrane pore formation. Dermcidin helps to limit bacterial colonization on the skin during the initial hours after sweating, and therefore effectively reduces the risk of infection by pathogens encountered in the environment. Notably, dermcidin is one of the principal antimicrobial components in sweat and is continuously supplied to the skin surface during perspiration. Other sweat antimicrobials include lysozyme, lactoferrin, and shorter peptides like RNase 7 and psoriasin, but dermcidin-derived peptides are especially abundant and critical for innate defense [25,26,27].

Clinical studies suggest that deficiencies in dermcidin may predispose individuals to skin infections. Patients with atopic dermatitis (AD), for instance, have significantly reduced levels of dermcidin peptides in their sweat [28]. This correlates with AD patients’ impaired innate skin defense and their susceptibility to recurrent Staphylococcus aureus and viral infections [29]. It has been shown that sweat from AD patients contained markedly lower dermcidin-derived peptide concentrations compared to healthy controls, particularly at sites of eczema lesions [22]. Similarly, low dermcidin expression has been noted in inflammatory acne, potentially permitting overgrowth of Cutibacterium acnes in sebaceous units [30]. On the other end of the spectrum, recent transcriptomic analyses in hidradenitis suppurativa patients have found dermcidin expression to be significantly downregulated in lesional skin, suggesting a possible link to disease pathogenesis [31]. These findings collectively indicate that dermcidin is a crucial component of the skin’s baseline antimicrobial barrier, and its insufficiency can undermine skin health.

### 2.2. Defensins

Defensins are a large family of cysteine-rich cationic peptides that serve as crucial antimicrobial effectors in many tissues, including the skin. Human defensins are arginine-rich AMPs, ranging in size from 2 to 5 kDa and sharing a conserved structural motif of three intramolecular disulfide bonds [8]. According to their disulfide arrangement, human defensins are generally categorized into alpha-defensins and beta-defensins [32]. Alpha-defensins have been primarily found in neutrophils and intestinal Paneth cells, while beta-defensins are predominantly produced by epithelial cells, in particular, by keratinocytes.

In the skin, the beta-defensins are especially important. Human β-defensin 1 (hBD-1) is constitutively expressed at low levels in normal skin, providing a constant background defense. In contrast, human β-defensin 2 (hBD-2) and β-defensin 3 (hBD-3) are typically inducible: their production is upregulated in response to infection, inflammation, or even ultraviolet light exposure.

Each defensin has a distinct spectrum of activities. HBD-2 was the first AMP isolated from the human skin, and it is the most effective against Gram-negative bacteria [33,34]. HBD-3 has a broader antimicrobial range that includes Gram-positive bacteria like *Staphylococci*, in addition to Gram-negative ones. HBD-3 is one of the most potent defensins that can kill antibiotic-resistant bacterial strains such as MRSA [35]. HBD-2 and HBD-3 are typically not present at high levels in healthy skin but can be strongly induced during episodes of skin inflammation or infection. For example, during a bacterial invasion or in psoriatic inflammation, keratinocytes ramp up HBD-2/HBD-3 expression, contributing to the relative resistance of psoriatic plaques to infection. UVB irradiation can also modulate HBD-2 and -3 expression, pointing to their role in photoprotection or post-UV immune surveillance [36].

In disease contexts, defensins exhibit contrasting patterns. Psoriasis is characterized by overproduction of β-defensins; psoriatic lesions contain high levels of hBD-2 and hBD-3, as well as other AMPs such as LL-37 and psoriasin [37,38]. This may help to conceive an explanation for why the psoriatic skin rarely gets infected despite breaches in the barrier. Indeed, genomic studies have found that some psoriasis patients carried extra copies of the β-defensin gene cluster, potentially leading to a higher peptide output [39]. However, these abundant AMPs might also stimulate the immune system: defensins can act as “alarmins” that activate immune cells, possibly contributing to psoriatic inflammation. By contrast, AD differs in an inadequate induction of defensins. The lesional AD skin has significantly lower hBD-2 and hBD-3 levels than those detected in other forms of dermatitis. Th2 cytokines IL-4 and IL-13, which are elevated in AD patients’ skin, actively suppress the defensin and cathelicidin expression in keratinocytes. The AMP deficiency in eczema patients partly underlies a high propensity of the AD skin to *Staphylococci* and viral infections. Interestingly, though baseline defensin levels are low in AD, if severe infection occurs, a subset of AD patients can upregulate defensins [40,41]. Thus, the exact role of defensin deficiency might vary between patient subgroups. Overall, defensins are indispensable for the normal skin defense, and therapeutic boosting of their levels or function is being explored for infection-prone conditions.

### 2.3. Cathelicidin LL-37

Cathelicidin LL-37 is another host defense peptide, notable for being the only cathelicidin-family member expressed in humans. The peptide is also known as the cathelicidin antimicrobial peptide (CAMP) [42,43,44]. It is encoded by the *CAMP* gene of the precursor protein termed the cationic antimicrobial peptide-18 (hCAP-18), consisting of the N-terminal cathelin domain and the C-terminal peptide region. Proteolytic cleavage of hCAP-18 by skin proteases of the kallikrein family releases the active 37-amino-acid-residue peptide known as LL-37. In its mature form, LL-37 adopts an alpha-helical structure that enables it to effectively disrupt microbial membranes. LL-37 exhibits a broad spectrum of antimicrobial activities against Gram-negative and Gram-positive bacteria, and displays antifungal action against *Candida albicans* and antiviral effects. The human genome contains only one cathelicidin gene, also known as human cationic antimicrobial peptide-18 (*hCAP-18*), cathelicidin antimicrobial peptide (*CAMP*), or leucine leucine-37 (*LL-37*) [45,46]. This peptide, synthesized initially as a propeptide, is cleaved to form cathelin and the C-terminal LL-37, known for its antimicrobial activity [47,48].

Under homeostatic conditions, the level of cathelicidin expression is low in the skin [49]. Keratinocytes in healthy skin produce LL-37 in small quantities. However, during infection, injury, or inflammation, cathelicidin production is strongly induced in keratinocytes and is also delivered to sites of concern by infiltrating neutrophils, which carry pre-formed LL-37 in their granules [50]. Thus, LL-37 is rapidly available at wounds or infection foci, where it not only directly kills microbes but also orchestrates immune responses. LL-37 is a prominent example of AMPs that doubles as an immune signal molecule. It can stimulate the release of chemokines and cytokines from a variety of cells and act as a chemoattractant to immune cells [47]. For instance, LL-37 can attract neutrophils, monocytes, T cells, and mast cells, helping recruit these cells to damaged or infected skin. It also triggers keratinocytes and mast cells to produce cytokines that amplify local immune responses. At a molecular level, LL-37 interacts with receptors like formyl peptide receptors and can modulate Toll-like receptor (TLR) signaling as well as P2X7 purinergic receptors on immune cells. Through these pathways, LL-37 influences processes such as inflammatory cascade activation, angiogenesis by stimulating endothelial cell proliferation, and even wound re-epithelialization [51].

Given its potent activities, it is not surprising that dysregulation of LL-37 is linked to skin diseases. In atopic dermatitis (AD), as mentioned, there is evidence for insufficient induction of cathelicidin: the AD skin often fails to upregulate LL-37 after injury, likely due to the inhibitory effect of Th2 cytokines. This contributes to the weakened antimicrobial barrier in eczema and chronic non-healing lesions. In psoriasis, conversely, cathelicidin LL-37 is overexpressed in lesional skin. One intriguing hypothesis is that in psoriasis, LL-37 forms complexes with self-DNA or self-RNA from damaged cells, and these complexes trigger plasmacytoid dendritic cells to produce interferon [46,47]. Essentially, an autoimmune inflammation loop is initiated by an AMP. Thus, it is still debated whether LL-37’s net effect is protective or pro-inflammatory in psoriasis. Rosacea also provides another twist: rosacea patients have normal or elevated cathelicidin LL-37 expression, but abnormal processing. In rosacea, excessive activity of skin proteases, such as kallikrein 5, cleaves cathelicidin into shorter peptide fragments that are more pro-inflammatory than LL-37 itself. These fragments can induce skin inflammation, vascular changes, vasodilation, telangiectasia, and even pustule formation characteristic of rosacea. Thus, cathelicidin exemplifies how a host defense peptide can contribute to pathology when its regulation is awry—either by being too low, as in some eczema cases, or by being improperly activated, as in rosacea [52].

Notably, LL-37 is regulated by external factors such as vitamin D. The vitamin D pathway directly induces the *CAMP* gene transcription [53,54]. First, vitamin D is converted to 1,25-dihydroxyvitamin D3, which binds to the vitamin D receptor (VDR), and then vitamin D response elements (VDREs) directly activate the *CAMP* gene [55]. This mechanism is realized in the human skin and immune cells which improves the epithelial barrier function [56]. Clinical studies have revealed a correlation between the LL-37 and vitamin D levels in serum and skin diseases [57].

UVB exposure can trigger local vitamin D synthesis in skin, thereby increasing cathelicidin production [38,54,58]. This is one reason why rosacea, often affecting sun-exposed facial skin, might flare with sun, as UV induces cathelicidin expression, which, given the abnormal processing in rosacea, leads to inflammation. Additionally, under conditions of low vitamin D or during winter, cathelicidin levels may drop [59,60]. Therapies targeting this pathway are promising: topical and systemic administration of vitamin D or its analogues have been shown to raise LL-37 levels in the skin and are associated with improved infection control in AD and accelerated lesion healing. For instance, oral vitamin D supplementation in atopic dermatitis patients increased the skin LL-37 level and was correlated with fewer skin infections [53,61]. In psoriasis, vitamin D analogues, like calcipotriol, provide effective treatments [62]. Interestingly, they reduce inflammation while simultaneously increasing the cathelicidin expression in psoriatic plaques. This suggests that vitamin D can help to normalize the function of LL-37, enhancing its antimicrobial benefit while mitigating its pro-inflammatory danger. This is a nuanced outcome that is in need of further investigation.

### 2.4. Lactoferrin, Lactoferricin and Other Lactoferrin-Derived Peptides

Lactoferrin is the iron-binding glycoprotein found in many bodily secretions, such as tears, saliva, milk, and neutrophil granules [63]. It is known for its antimicrobial action and immunomodulatory functions [64,65,66,67]. Lactoferrin is also found in sweat and on the epidermal surface of the skin, where it contributes to the innate defense [68]. Proteomic analyses of human sweat have identified lactoferrin alongside highly abundant defense proteins, dermcidin, psoriasin, and others [69,70]. Its presence in sweat implies a role in protecting the skin against microbes by sequestering iron needed by microbes and directly interacting with pathogens. Indeed, sweat lactoferrin likely helps to defend eccrine-rich areas from infection. By methods of immunohistochemistry, lactoferrin has been detected in sweat gland cells, and it has been shown to be released onto the skin similarly to dermcidin. These findings suggest that lactoferrin is a part of the skin surface’s antimicrobial arsenal, especially under conditions of sweating.

When lactoferrin is enzymatically digested, for example, by pepsin in the stomach or proteases in inflamed tissues, its peptide fragments with enhanced antimicrobial activities can be released [71,72]. One of such peptides is lactoferricin, which corresponds to a segment of the N-terminal region of lactoferrin: in humans—the 49-amino-acid-residue fragment, and in bovines—the 25-residue cyclic peptide lactoferricin B. Remarkably, lactoferricin is much more potent as an antimicrobial agent than the parent lactoferrin protein. Discovery of lactoferricin’s superior activity led to its further extensive research as a potential therapeutic agent [73,74,75,76]. For instance, the bovine lactoferricin is effective at micromolar concentrations against a broad range of bacteria, including particularly sensitive Gram-positive bacteria. The peptide has membrane-disruptive effects and can also penetrate bacterial membranes and disrupt microbial cells. Lactoferricin adopts a beta-sheet structure in solution, which appears to facilitate its interaction with bacterial membranes and might partly explain why it outperforms full lactoferrin. Human lactoferricin forms a coiled structure [77]. Structural differences in lactoferricin and lactoferrin likely underlie the peptide’s greater microbicidal power.

For skin health, lactoferrin and lactoferricin offer multiple benefits. Their direct antimicrobial actions include activities against common skin pathogens like *Staphylococcus aureus* and fungi [76]. They also can disrupt biofilms and exhibit anti-biofilm properties, which is relevant in chronic wounds or acne lesions where biofilms impede treatment [65,66,67]. Additionally, lactoferrin exerts anti-inflammatory effects: it can bind and neutralize bacterial components such as LPS, reducing inflammatory signaling [78]. Lactoferrin is known to modulate immune responses, for instance by lowering excessive IL-6 and TNF-α production and promoting an appropriate inflammatory resolution [78]. This anti-inflammatory capability suggests lactoferrin or its peptides might help under conditions of inflammaging by mitigating some inflammatory mediators.

Clinically, lactoferrin has been investigated as a therapeutic supplement for skin medical care. In controlled trials, oral lactoferrin supplementation led to reductions in acne lesions, likely by reducing Propionibacterium (Cutibacterium) acnes proliferation and inflammation [79]. It has also been reported to aid tinea pedis and even plaque psoriasis and atopic dermatitis [80]. One systematic review concluded there is encouraging evidence that lactoferrin may be beneficial in acne, psoriasis, and for enhancing wound healing in diabetic ulcers [81]. Topically, lactoferrin has moisturization and barrier-supporting effects on the skin, potentially improving dryness and texture [82]. All these effects make sense given lactoferrin’s multifaceted actions: antimicrobial, keeping dysbiosis in check, anti-inflammatory, and even directly promoting skin barrier homeostasis. In some cases, researchers indicate that lactoferrin can stimulate dermal fibroblasts and re-epithelialization [83,84]. Human lactoferricin itself is being pursued as a therapeutic peptide.

### 2.5. Strategies for Maintaining Balance of Host Defense Peptides

Both deficit and excess of host defense peptides can be problematic, so the goal is to maintain a healthy balance—enough to protect against infection and dysbiosis, but not so much or in such form that it does not drive inflammation. Several novel therapeutic strategies are being explored in this regard, as described below.

#### 2.5.1. Application of Vitamin D and Its Analogues

Application of vitamin D is one well-established approach to enhance endogenous AMP production [53,54,58]. Vitamin D directly upregulates the gene expression of cathelicidin (LL-37) in keratinocytes and other cells. Ensuring adequate vitamin D levels through safe sun exposure or via oral administration can increase LL-37 output and potentially other AMPs, strengthening the skin’s antimicrobial barrier. In clinical studies, oral vitamin D administration has been shown to induce cathelicidin production in atopic dermatitis patients and was associated with fewer skin infections [40]. Topical calcipotriol, a vitamin D analogue, is used in psoriasis and, interestingly, may help to normalize AMP levels in psoriatic lesions, both reducing inflammation and increasing LL-37 and β-defensin expression levels, thereby restoring a more balanced immune state [85]. Thus, leveraging the vitamin D pathway is a precision tactic to adjust AMP levels: increasing them in conditions of deficiency or potentially reducing their inflammatory consequences. For example, inhibiting vitamin D activation might be beneficial in rosacea.

#### 2.5.2. Targeting Protease Activity

In diseases like rosacea or atopic dermatitis, aberrant protease activity either over-produces inflammatory peptides or degrades protective ones [86]. New therapeutic strategies aim to modulate the activities of such enzymes. For example, rosacea patients are characterized by an enhanced kallikrein 5 activity. This protease converts LL-37 into pro-inflammatory fragments, thus inhibiting its generation, which helps to prevent the generation of those harmful peptides, thereby reducing inflammation [52]. In atopic dermatitis, *Staphylococcus aureus* proteases can degrade AMPs, in particular LL-37. Reducing *S. aureus* colonization or using protease inhibitors in emollients could preserve host AMPs [87,88]. These approaches essentially seek to protect host defense peptides from inappropriate activation or destruction, maintaining their beneficial functions.

#### 2.5.3. Boosting of AMP Induction via TLR Agonists

Human epidermis serves as a natural barrier between the internal and external environment, where keratinocytes play an essential role [89]. Certain stimuli can trigger keratinocytes to ramp up AMP production. For instance, activation of Toll-like receptors (TLRs) in keratinocytes (in particular, TLR1 and TLR2) can induce β-defensins and cathelicidin LL-37 production [90,91,92]. Topical treatments containing TLR agonists or commensal microbe products activating TLR signaling pathways are under investigation. Enhancing local AMP expression without causing overt inflammation is a new therapeutic strategy for managing skin diseases. A compelling example is provided by the skin commensal *Staphylococcus epidermidis*. A small lipopeptide secreted by *S. epidermidis* has been shown to activate TLR2 in keratinocytes, inducing hBD2, hBD3, and LL-37 production [93]. Harnessing such commensal-derived signals might boost the skin’s innate defense in a controlled way. In fact, normal *S. epidermidis* might be used to trigger TLR2 and enhance AMP expression in skin, thereby preventing disease development.

#### 2.5.4. Synthetic AMP Mimetics and Peptide Therapeutics

A number of pharmaceutical efforts focus on creating synthetic peptides or peptidomimetics that replicate the action of natural AMPs [94]. Some of them are being developed as topical agents for acne treatment, wound healing, or fungal infection therapy. For example, omiganan is the synthetic cationic peptide that has undergone trials as a topical gel for rosacea and acne care, aiming to reduce microbial triggers of inflammation [95]. While not a human or marine peptide per se, it exemplifies the strategy of supplying an AMP externally to bolster the skin’s defenses. Another approach involves the use of stabilized analogues of human AMPs with modifications that make them more resistant to degradation in the skin [96]. By applying such peptides, one can locally compensate for a patient’s deficiency in AMP production. This strategy is closely related to “replacement therapy” for AMPs.

#### 2.5.5. Selective AMP Modulation (Precision Medicine)

Main challenges in the therapeutic use of AMPs are avoiding a disbalance of the beneficial microbiota and preventing excessive inflammation. Precision strategies are being explored to specifically target harmful microbes or inflammatory pathways. One innovative concept is engineering so-called precision-guided AMPs fused to a targeting domain that directs them to a specific bacterium without effect on the resident microbiota [97]. In this scenario, the peptide kills the pathogen of interest and spares commensals. Such targeted AMPs might, for instance, selectively eliminate from the skin the common eczema pathogen *S. aureus* without wiping out *S. epidermidis* and other beneficial microbes. This approach has been demonstrated with a conjugated AMP that specifically modulated the microbiome, removing disease-causing species and improving outcomes. Using bioinformatics and AI, it is possible to design peptides that specifically interfere with inflammatory pathways. Databases and predictive algorithms allow the identification of peptide sequences likely to have anti-inflammatory activity [98]. Using these tools, scientists have recently identified novel fish-derived peptides suppressing inflammatory cytokine production and oxidative stress. These peptides might be developed into pharmaceuticals that could slow down “inflammaging”—the aging-related inflammation—by precisely decreasing excessive inflammatory signals combined with antimicrobial benefits.

#### 2.5.6. Lifestyle and Barrier Support

Maintaining host defense peptides balance includes not only direct medical interventions, basic skincare and lifestyle matters. Too dry or pH-disrupted skin can impair AMP function [99]. Mild acidification of the skin, using pH-appropriate cleansers, can optimize conditions for the biological action of AMPs like defensins and dermcidin, which are most active at acidic pH [100]. Avoiding harsh soaps and irritants can prevent the removal of natural AMPs from the skin surface or damage to AMP-producing cells. There is evidence that regular exercise may actually benefit the skin’s microbial defense by depositing dermcidin and other sweat AMPs in the skin consistently [101]. This is a natural way to recharge the antimicrobial shield. Nutrition can also play an important role: diets enriched in vitamin D and other micronutrients support the body’s ability to produce AMPs [53,54,57]. These supportive measures, while indirect, contribute to an environment where host defense peptides can function effectively.

In summary, a variety of strategies, including vitamin D supplementation, probiotics use, and advanced peptide engineering, are converging to sustain optimal levels and activity of host defense peptides in the skin. Success in these approaches holds promise not only for preventing infections but also for controlling inflammation and maintaining a balanced relationship with the skin microbiome.

## 3. Marine Host Defense Peptides for Skin Disease Prevention and Protection

Marine organisms inhabit environments where bacteria comprise 70% of their total biomass, and therefore have robust defenses that include antimicrobial peptides [16]. Marine AMPs have been found to often contain unusual amino acids, cyclic backbones, and multiple disulfide bonds [102]. They display a broad spectrum of biological activities, ranging from antibacterial and antifungal to anti-inflammatory and anticancer. Furthermore, many marine peptides are effective against drug-resistant pathogens and can act at high salt concentrations and extreme pH levels [102]. This makes them attractive candidates for the development of new dermatological treatments, particularly in the context of declining efficacy of traditional antibiotics.

### 3.1. Antibacterial Peptides from Marine Organisms

Mechanisms of action of most marine AMPs are based on the destruction of bacterial cell walls and membranes [103]. This mode of action is advantageous because it is difficult for bacteria to develop resistance to membrane-targeting agents, as this needs to change fundamentally the electrophysiological properties of membranes. Additionally, some marine AMPs can bind bacterial endotoxins (LPS), thereby neutralizing inflammatory triggers while killing the microbes [104].

A classic example of a marine AMP is a family of fish peptides, piscidins. These AMPs are ribosomally synthesized, are often found in fish gill and skin immune cells, and are homologous to cathelicidins. One member, pleurocidin, was first isolated from the skin mucus of the winter flounder *Pleuronectes americanus* [105,106]. Pleurocidin exhibits potent antibacterial effects against both Gram-negative bacteria, such as *Pseudomonas* and *Vibrio* species, and Gram-positive bacteria, like *Streptococcus* [107]. It helps to protect fish from infections in the marine environment, and in lab studies, pleurocidin has been shown to display activities against human pathogens as well. Notably, pleurocidin retains its activity at high salt concentrations comparable to those in seawater or sweat. In fact, fish-derived AMPs, for example, epinecidin, remain effective at salt concentrations that inactivate human AMPs like beta-defensin-1 [108,109]. Such a salt tolerance might be useful for skin applications, since human sweat and wound exudates are relatively saline, where some human AMPs become inefficient.

Another well-studied marine peptide is arenicin, isolated from the marine lugworm *Arenicola marina* [110,111]. Arenicin is a unique β-hairpin peptide enriched in arginine and tryptophan residues [112,113,114]. It has shown potent activities against both Gram-positives and Gram-negatives, including resistant strains. Arenicin works by permeabilizing bacterial membranes and possibly interfering with intracellular targets. Interestingly, arenicin and its analogues can also bind lipopolysaccharide, reducing LPS-induced inflammation at sub-lethal concentrations [112,113,114]. This means that arenicin can simultaneously kill bacteria and reduce endotoxic shock or inflammation, which is a valuable dual action for infected skin wounds or ulcerative lesions.

In general, marine polychaeta are an abundant source of novel host defense peptides. The peptides capitellacin and abarenicin have been isolated from *Capitella teleta* [115,116,117] and *Abarenicola pacifica* [118], respectively. Capitellacin effectively acts against a wide panel of bacteria via a non-lytic mechanism. Abarenicin displays a pronounced activity against Gram-negative bacteria, including drug-resistant ones, and acts on different steps of the biofilm formation. By contrast with capitellacin, abarenicin disrupts bacterial and mammalian membrane integrity. Considering its antibacterial potential, abarenicin was selected for further modification by replacing individual amino acid residues in order to improve its membrane selectivity. This allowed us to design the Ap9 analog, displaying a high efficacy in the septicemia and neutropenic mice peritonitis models. A low cytotoxicity towards human cells and high selectivity make both peptides promising candidates for the development of a novel anti-infective drug. Besides, neither peptide resembles human ones, which allows for avoiding cross-reactivity and side effects.

Marine crustaceans and mollusks also contribute to antimicrobial agent candidates. In marine invertebrates like mussels, peptides such as mytilins and defensins have been characterized as strongly inhibiting *Staphylococcus aureus* and other skin-relevant bacteria [119,120]. The horseshoe crab *Tachypleus tridentatus* provided one of the first discovered marine AMPs, tachyplesin, which strikes a broad range of bacteria and some fungi [121]. Tachyplesin’s robust antibacterial activity inspired the design of numerous synthetic analogues [122]. This exemplifies how marine peptides can serve as templates for the bioengineering of novel drugs.

Epinecidin-1 from the orange-spotted grouper fish has been shown to be effective against 24 bacterial strains, including multidrug-resistant *Staphylococcus aureus* and *Pseudomonas aeruginosa*, with minimum inhibitory concentrations (MICs) comparable to conventional antibiotics [108]. In mouse models of wound infection with MRSA and *P. aeruginosa*, epinecidin-1 significantly reduced bacterial counts and improved survival without a notable toxicity [109,123].

In practical terms, several marine AMPs are being investigated as topical antimicrobials to prevent or treat skin infections. Due to their ability to damage the membrane and act upon different microbial targets, marine AMPs represent promising drug leads to combat antibiotic-resistant skin infections.

### 3.2. Marine Peptides with Antifungal Activity

Antifungal action of marine peptides is mainly based upon disrupting membranes, but some of them can bind to hitin or lipids of fungi. For instance, mytimycin from mussels and penaeidins from shrimps have shown a pronounced activity against *Candida albicans*, the common yeast that can infect skin folds and wounds [124,125]. Dolastatins, originally isolated from the sea hare *Dolabella auricularia,* but later found in symbiotic cyanobacteria, have shown a high antifungal activity against the pathogenic yeast *Cryptococcus neoformans* along with antiproliferative anti-cancer properties [126,127].

Dolastatins target microtubules, manifesting a unique intracellular mechanism rather than direct membrane lysis. While dolastatins are more famous as anticancer leads, their antifungal efficacy indicates the untapped potential of marine peptides.

Halocidin from the tunicate *Halocynthia* has been found to display strong fungicidal effects [128]. Synthetic analogues of halocidin kill fungi of *Candida* species by presumably forming pores in fungal membranes. Halocidin analogues, in particular, their dimeric form (di-K19Hc), were able to eradicate *Candida albicans* in vitro at low micromolar concentrations [129]. It would appear that halocidin-derived peptides might be developed into topical antifungal agents, especially for drug-resistant *Candida* or for fungal biofilms that are recalcitrant to azoles.

Marine fish peptides also contribute to antifungal defense. Mentioned above, piscidins are not only antibacterial peptides but can also inhibit fungi [130]. Some piscidins and their synthetic derivatives have been shown to kill *Candida albicans* by permeabilizing its cell membrane. The grouper AMP epinecidin-1 is active against both bacteria and fungi. This peptide has demonstrated antifungal effects in agricultural models—it suppressed the growth of *Botrytis cinerea*, the mold that causes a gray rot on harvested fruits. It has been shown that treatment of peaches with epinecidin-1 prevented a gray mold spoilage, highlighting its antifungal potency in a real-world setting. It is expected that marine AMPs might help to protect human skin from fungi as well.

Marine cyanobacteria from blue-green algae also produce antifungal peptides. Hassallidin A from the cyanobacterium *Hassallia* is a glycosylated lipopeptide that exhibits a potent antifungal activity against *Aspergillus fumigatus* and *Candida albicans* [131,132]. It likely inserts into fungal membranes, aided by its lipid tail and sugar moieties, which causes a leakage of fungal cell contents.

These examples illustrate that marine peptides can serve as broad-spectrum antimycotic agents, effective against dermatologically relevant fungi. They might be especially useful against fungi that have developed resistance to standard antimycotics, like azole-resistant *Candida* or *Trichophyton* species in chronic athlete’s foot. A hurdle in crossover them to human skin or nail fungi treatments is ensuring that they penetrate the skin or nails and remain stable in the milieu. Lipopeptides may inherently tend to penetrate skin due to their amphiphilic nature.

In conclusion, marine host defense peptides hold considerable promise as antifungal agents. By interacting with fungal membranes or critical intracellular targets, these peptides could complement or replace traditional antimycotics. Marine AMPs may represent the next generation of antimycotic agents with novel mechanisms to combat yeast and mold infections of the skin.

### 3.3. Wound Healing

During wound healing, regenerative processes can be inhibited by infection and excessive inflammation. Host-derived AMPs can exert antibacterial activity and attract immune cells to the site of injury. Debrided wounds tend to have reduced bacterial counts but also show improved granulation tissue formation and re-epithelialization rates [133]. While detailed mechanisms of wound healing are still under investigation, it has been shown that epinecidin-1 could stimulate keratinocyte and fibroblast migration, important for closing of the wound, and may induce growth factors or chemokines that orchestrate the repair [123,134,135,136,137,138]. Moreover, the epinecidin-1 ability to temper excessive inflammation by killing bacteria may otherwise trigger intense inflammatory responses. It means that wound healing can proceed through normal stages without a prolonged inflammatory phase. A balanced inflammatory response is important: subinflammation might not clean the wound of debris, but a severe inflammatory process may cause tissue damage. AMPs help strike that balance by acting as natural immunomodulators.

Marine peptides have also been incorporated into biomaterial dressings for wounds. For instance, peptide-infused hydrogels or nanofibers that continually release AMPs can be applied to a wound [139,140,141,142]. Such dressings could keep the wound sterile while also directly interacting with cells to promote healing. An example is the use of the fish collagen peptide scaffold that not only provides a matrix for cells to grow but also contains peptides that reduce inflammation and oxidative stress, thereby fostering better healing [143,144,145]. Marine collagen from fish skin or scales is used in some wound dressings; it releases collagen peptides, which have chemotactic effects on fibroblasts and can encourage new collagen deposition in the wound bed [146]. It has been found that fish collagen peptides could speed up wound closure and improve the strength of the healed skin, for one part, by acting as a scaffold and, for the other part, by signaling the body to ramp up its repair processes [147,148].

Another benefit of AMPs in wounds is their effect on immune cells like macrophages. Normally, in wound healing, macrophages transition from a pro-inflammatory “M1” state in early stages, for example, required to kill microbes, to an anti-inflammatory “M2” state in later stages, needed to promote tissue repair. Chronic wounds often get stuck with macrophages in the M1 state, spewing inflammatory cytokines and proteases that degrade new tissue [149,150]. AMPs can help to push macrophages toward the M2 phenotype. For example, certain peptides can directly act on macrophages to induce the expression of growth factors and to reduce pro-inflammatory TNF-α, thus encouraging the wound to move into the proliferation phase. Marine peptides with immunomodulatory capacity, like the algae-derived peptide PPY1, which inhibits pro-inflammatory cytokines, could be very useful if delivered into a wound, calming excessive inflammation.

Recently, it has been found that many AMPs, including marine-sourced ones, promoted wound healing by a synergistic mechanism: they provided an emergent control over infection and actively regulated immune cells, thus supporting tissue regeneration.

### 3.4. Anti-Inflammatory Properties of Marine AMPs

Marine AMPs realize their anti-inflammatory properties through the following mechanisms.

#### 3.4.1. Neutralizing LPS from Bacteria

Certain fish peptides have LPS-binding ability and can reduce LPS-induced cytokine release. In a skin context, this means that even if bacteria are present, an AMP could dampen the “danger signals” they emit, thereby preventing a full-blown inflammatory cascade [151].

#### 3.4.2. Direct Cytokine Suppression

Some marine-derived peptides have been shown to suppress the production of pro-inflammatory cytokines and mediators. The thalassospiramides A and D from a marine bacterium, for instance, were found to inhibit IL-5 expression and NO production in immune cells, suggesting utility in Th2-mediated inflammatory conditions like asthma or allergic dermatitis [152]. The peptide PPY1 from the red algae *Pyropia yezoensis* completely inhibits LPS-stimulated release of NO and significantly reduces levels of iNOS, COX-2, IL-1β, and TNF-α in activated macrophages [153]. Such a broad inhibition of inflammatory mediators indicates a powerful anti-inflammatory potential. If applied to an inflammatory skin lesion, PPY1 or similar peptides might reduce redness, swelling, and collateral tissue damage by lowering those cytokine levels. Indeed, PPY1 has been suggested as a potential drug for the treatment of inflamed skin.

#### 3.4.3. Antioxidant Effects

Peptides from different species of marine organisms, from algae to fish, demonstrated an antioxidant effect [154,155,156,157]. By scavenging reactive oxygen species, these peptides indirectly reduce oxidative damage and the subsequent inflammatory signaling that damage might cause [158,159]. For instance, antioxidant peptides from tuna and abalone have been shown to neutralize free radicals and reduce UV-induced cell damage, which in turn diminished the inflammatory response to UV radiation [160]. Less oxidative stress means less noticeable activation of pathways like NF-κB that drive inflammation.

Due to these modes of action, marine peptides are being investigated for the development of therapeutic strategies to treat inflammatory skin diseases such as psoriasis, atopic dermatitis, and rosacea. Unlike corticosteroids or calcineurin inhibitors, which broadly suppress immunity with side effects and risk of infection, AMP-based therapies could selectively curb harmful inflammation while still allowing normal immune surveillance. For example, the AI-designed peptide derived from a mesopelagic fish has been identified. The peptide specifically displayed anti-inflammatory activity without notable toxicity. This peptide QCPLHRPWAL has been predicted and then confirmed to reduce inflammatory markers and showed analgesic activity in experimental animal models [161]. One could imagine such a peptide being used in a cream for mild eczema to reduce itch and redness by targeting the inflammatory pathways, possibly with less risk than a steroid.

### 3.5. Inflammaging

Aging skin often is characterized by an uptick in colonization by pro-inflammatory microbes, for example, an overgrowth of *Staphylococcus aureus* cell counts or a decrease in beneficial *Staphylococci* that normally keep inflammation in check [162,163]. By using targeted AMPs that specifically remove so-called “bad actors”, one could reduce the chronic immune activation that they cause. For instance, a precision-guided AMP could be designed to seek out and kill *S. aureus* in the skin of an elderly person, thereby lowering the subclinical inflammation load, but spare the commensals that might even produce their own beneficial anti-inflammatory factors. A proof-of-concept for this exists in the nasal microbiome: a targeted peptide has been used to eliminate a pathogen and it shifted the microbial community to a healthier state [164,165]. This demonstrates that such precision antimicrobials are feasible. Similarly, in skin, a targeted AMP could maintain a youthful microbiome composition, indirectly reducing inflammaging.

Another strategy is direct cytokine targeting by peptides. Inflammaging is characterized by elevated IL-1, IL-6, TNF-α, and other cytokines even in the absence of overt infection [166,167,168,169]. Some marine peptides or their derivatives might be able to bind these cytokines or block their receptors in a highly specific way. While most of above- mentioned peptides act upstream, preventing the release of cytokines, one could also envision peptide-based inhibitors of cytokine signaling that might influence the receptor-ligand interactions of inflammatory mediators. These could be delivered in a targeted way, for example, being encapsulated into nanoparticles that would home to inflamed skin [170]. Such nanoparticles can be functionalized with antibodies or ligands for inflamed endothelium [171]. In fact, modern research in nanotechnology is centered on AMP delivery systems that release peptides in response to specific triggers, like an acidic pH in an inflamed tissue. This ensures that the peptide exerts its action primarily towards its target, minimizing systemic exposure.

Precision in dosing and structure is also crucial. Marine peptides can be engineered to enhance certain activities [172,173,174,175,176]. For instance, make an AMP more anti-inflammatory and less cell-toxic by the substitution of a few amino acids. Using computational design, one can now design weak AMP sequences for desired properties. For inflammaging, one might sacrifice some direct antimicrobial potency in exchange for stronger LPS-binding or a higher affinity for TLR. In this case, the peptide’s main role would become soaking up inflammatory stimuli rather than killing bacteria. These second-generation peptides could be delivered at low doses over long periods to provide a continuous dampening of microinflammation, thereby slowing collagen degradation and cellular senescence induced by chronic inflammation.

Moreover, some marine peptides can specifically target senescent cells, having a senescence-associated secretory phenotype known as SASP [177]. Those cells are accumulated with age and characterized by high levels of inflammatory cytokines and other immune factors [178,179]. There is emerging interest in peptide-based senolytics or senomorphics—specific agents that either kill senescent cells or suppress their inflammatory secretions. Though these compounds are not yet well-developed, marine natural sources have yielded some candidate products that affect aging pathways. It has been found that the peptide that could enter senescent fibroblasts in skin modulate their output, perhaps via turning on the stress response factor Nrf2, and decrease the level of inflammatory cytokines [156,180]. This marine peptide might be a boon for anti-inflammaging therapy.

Finally, precision strategies also encompass combination approaches based upon the use of marine peptides alongside other treatments in a complementary way. For example, combining a marine AMP with low-dose antioxidants could address both oxidative stress and inflammatory signaling in aging skin [181]. Another concept is using an AMP cream in the day to keep the microbiome balanced and an unrelated anti-aging compound at night, achieving multi-targeted prevention of skin aging [182].

To sum up, the goal of precision use of marine host defense peptides in inflammaging is to intervene in specific pathways that link aging, microbe growth, and inflammation development. By carefully selecting or designing peptides that neutralize microbial triggers, modulate immune receptors, or correct age-related immune dysregulation, it is possible to reduce smoldering inflammation that drives aging. This could translate to visible benefits—less redness, improved texture, slower wrinkle formation, and, more importantly, healthier skin function, including better barrier, faster repair, lower incidence of infections or lesions in the elderly.

Although many of these strategies remain in early research phases, the underlying concept is compelling.

## 4. Marine and Human Host Defense Peptides for Slowing Skin Aging

Skin aging is influenced by environmental stressors, including UV radiation and natural decline in cellular function [183,184,185,186,187,188,189]. Marine peptides overlap with host defense in a broad sense—protecting cells and tissues from damage, microbes, oxidative stress, or UV light [154,158,190,191,192,193]. The cosmeceutical industry has already started to incorporate marine peptides, for example, algae ones, into products, often boasting improved skin firmness or wrinkle reduction. Here we tie those applications to the scientific findings that underlie them.

### 4.1. Anti-Aging Effects

Marine peptides contribute to anti-aging effects in the skin via several pathways: enhancing collagen synthesis, protecting collagen and elastin from breakdown, providing antioxidants to counteract free radical damage, and maintaining hydration and barrier function [193,194,195,196].

One of the most prominent marine-derived ingredients in anti-aging skincare is marine collagen peptides. Typically derived from hydrolysates of fish skin or scales or from marine invertebrates, these are represented by peptides rich in glycine, proline, and hydroxyproline—the major amino acid residues in human collagen [143,144,145,146].

Marine peptides also display antioxidant activities, as many of them are able to scavenge free radicals or chelate metal ions that catalyze oxidative reactions. For instance, the peptides ICRD and LCGEC derived from tuna eggs exhibited a strong DPPH radical scavenging activity and protected skin cells from oxidative damage [180]. By neutralizing free radicals, these peptides help to prevent one of the major causes of skin aging: oxidative stress that leads to cellular senescence and breakdown of the dermal matrix. Similarly, three small peptides (PKK, YEGGD, GPGLM) isolated from the skipjack tuna have been found to effectively eliminate ROS and reduce lipid peroxidation inside cells [197,198]. This demonstrates that marine peptides can bolster the human skin antioxidant defenses, which typically wane with age. Antioxidant peptides complement traditional antioxidants like vitamins C and E, possibly by reaching corresponding cellular compartments or regenerating other antioxidants.

Another target in anti-aging is matrix metalloproteinases (MMPs)—the enzymes breaking down collagen and elastin [199]. UV exposure and inflammaging increase MMP levels, leading to wrinkles and loss of elasticity [200]. Some marine peptides have shown the ability to downregulate MMP expression or directly inhibit their activities. For example, certain seaweed-derived peptides could inhibit MMP-1 expression in fibroblasts, thereby protecting collagen from degradation [201]. Additionally, mycosporine-like amino acids (MAAs) from marine algae are not peptides, but sometimes are included in peptide-based formulations [156,202,203,204]. MAAs can absorb UV and prevent photo-damage [205]. In synergy, MAAs and antioxidant peptides provide a shield against environmental aging factors.

Some marine peptides have a hydrating and barrier-stabilizing effect. Lactoferrin, when applied to skin, has been found to improve the skin barrier and moisture content [206]. Moisturized skin is less prone to forming fine lines and microcracks. Some fish peptides can induce the production of natural moisturizing factors or increase the expression of proteins like filaggrin that fortify the stratum corneum [207,208]. A trial of lactoferrin cream in winter has shown that consumers had improvements in skin moisture and texture, suggesting systemic benefits on skin health, presumably due to both immune modulation and direct skin effects [81].

Recently, it has been shown that application tests of the recombinant conotoxins TIIIA and TIIIAlaMut demonstrated their potential in cosmeceuticals, particularly in improving skin texture and tone and reducing periocular wrinkles [209].

Evidence is emerging that marine peptides can extend cellular lifespan or at least health span. A protein hydrolysate from the sea cucumber has been shown to have anti-aging effects in mice and fruit flies, where it improved antioxidant status and reduced markers of aging [210]. This hydrolysate, containing various peptides, presumably activated stress response pathways that promote longevity. Upregulating antioxidant enzymes or activating AMPK effects have been shown with sea cucumber peptides modulating NRF2/AMPK. If similar exposure applies to human skin cells, such peptides could maintain a more youthful phenotype in fibroblasts and keratinocytes, delaying senescence and functional decline.

### 4.2. Anti-Photoaging Activity of Marine Peptides

Mechanisms of photoaging include direct DNA damage by UV, generation of reactive oxygen species, inflammation, and upregulation of MMPs degrading collagen [211]. Marine host defense peptides and other bioactive marine peptides have shown considerable promise in protecting the skin from photoaging by addressing these mechanisms, functioning as natural sunscreens, antioxidants, and cellular protectants [197].

Some marine peptides can directly absorb UV radiation or dissipate its energy [160,210]. Apart from peptides per se, certain small mycosporine-like amino acid (MAA) compounds possess properties of UV-absorbers. MAAs are secondary metabolites discovered as natural photoprotectors in different marine organisms [155,160,193,202,210]. Their biotechnological applications, in particular, in the development of next-generation cosmetics with UV-absorbing properties and efficient against UV damage, are of much interest [212]. Peptide conjugates, which might deliver such UV-absorbing compounds to the skin, are being explored. However, a more practical scenario is the application of peptides that combat the aftermath of UV exposure, including oxidative stress and DNA damage.

As mentioned earlier, several marine peptides have potent antioxidant activities. Tuna egg-derived peptides (ICRD and LCGEC) have demonstrated the ability to protect human keratinocytes (HaCaT cells) from UVB-induced damage [158]. They efficiently scavenged free radicals (like DPPH radicals) and thereby reduced the oxidative stress load on cells after UVB irradiation. This results in lower lipid peroxidation and preservation of cellular components.

The abalone peptide, ATPGEG, has been shown to significantly mitigate UVB-induced reactive oxygen species (ROS) in skin cells and inhibit UVB-triggered DNA damage [159]. UV irradiation often causes specific DNA lesions. By reducing ROS and possibly enhancing DNA repair, the peptide might help to maintain genomic integrity in skin cells, preventing mutations and photoaging signs.

Antioxidant peptides from marine sources also reduce UV-induced inflammation. UV exposure typically leads to erythema through an inflammatory response. By neutralizing ROS and inflammatory intermediates, marine peptides can reduce the secretion of IL-6 and IL-8 that contribute to the sunburn reaction and post-UV matrix degradation [155,156,213].

Another pathway of photoaging is melanin overproduction, leading to age spots and uneven tone. Some marine peptides have been found to influence melanogenesis [211,214,215]. For example, the peptide from the pearl oyster *Pinctada martensii* inhibits tyrosinase (TYR), a major enzyme in melanin synthesis, hinders UV-induced hyperpigmentation, and contributes to a more even skin tone [216]. Skin-lightening peptides from fish or algae also might be valuable in preventing or fading sun spots.

Marine peptides also help to maintain the extracellular matrix after UV exposure. UV upregulates production of MMP-1, -3, and -9, which break down collagen and elastin [190]. Anti-photoaging peptides from tuna have been shown to downregulate these MMPs or upregulate TIMPs, thereby preserving collagen [217]. Also, by reducing ROS, they indirectly prevent the activation of AP-1, a transcription factor that increases MMP expression under UV stress.

Marine peptides can be incorporated into sunscreens and anti-aging creams to specifically prevent photoaging and inflammation. These peptides complement the UV filters by providing a biological line of defense. Once UV has penetrated, the peptides get to work repairing and mitigating the damage. When conventional treatments fail, marine peptides may provide benefits that standard sunscreens, which only block UV, cannot provide, such as mopping up radicals or calming post-UV inflammation. For example, the boiled abalone extract peptide ATPGDEG has been tested and found to have antiphotoaging effects in UVB-irradiated skin cell models, reducing markers of stress in those cells [218]. This reinforces that unique peptides from marine organisms can be harnessed as protective compounds.

Seaweeds are another rich source of bioactive peptides [193,194,195,201]. Certain brown seaweed or red algae peptides can induce the Nrf2 pathway in skin cells, which leads to increased production of endogenous antioxidants like glutathione and heme oxygenase-1 [219]. Activation of Nrf2 is a master switch to protect skin from UV-induced oxidative damage, and if marine peptides can trigger this pathway, they might effectively increase the skin’s resilience to UV stress.

Thus, marine host defense peptides are valuable allies in the fight against photoaging due to their:

- antioxidant capacity by neutralizing UV-generated ROS;
- DNA protection via reducing UVB-induced DNA damage in skin cells;
- anti-inflammatory action by curtailing the inflammatory response to UV and thus lessening redness and collagenase activity;
- tyrosinase inhibition that results in preventing overproduction of pigment, leading to more uniform skin after sun exposure;
- induction of collagen synthesis.

## 5. Influence of Microbiome Peptides on Skin Senescence

The human skin hosts a broad community of microorganisms. The major bacterial components of the healthy skin flora subsume *Corynebacterium*, *Propionibacterium,* and *Staphylococcus* species. The skin successfully coexists with commensal microorganisms, ensuring its safety from the harm of dangerous pathogens. Disturbance of this balance, known as dysbiosis, may be derived from a shift in the skin bacteria proportion and/or a changed response of the immune system to them, which may lead to inflammation and skin diseases. Novel therapeutic agents normalizing the skin microbiota ecology might provide an efficient instrument to dermatologists for the treatment of skin dysbiosis [220].

The skin microbiome undergoes changes with age, and these changes can either mitigate or exacerbate skin aging and senescence. For instance, a diverse and balanced microbiome can help maintain youthful skin functions by training the immune system and preventing pathogen colonization [221]. Dysbiosis contributes to a low-level chronic inflammation (inflammaging) and impairs the barrier function in older skin [162,222].

In this context, microbiome peptides refer to compounds that either are produced by skin microbes or modulate the microbial community. Skin commensal bacteria produce their own antimicrobial peptides to compete with rivals, and these peptides can incidentally protect the host by killing pathogens [223]. On the other hand, host defense peptides assemble the microbial community thriving in the skin, thereby maintaining a dynamic interplay.

Skin aging is accompanied by profound structural, functional, and immunological changes, many of which intersect with alterations in the skin microbiome [222]. Chronological aging and photoaging are associated with reduced microbial diversity, changes in dominant species, and altered microbial metabolite profiles. These changes can either mitigate or exacerbate senescence-related processes [168]. A diverse and balanced microbiome supports epidermal renewal, maintains antimicrobial defense, and suppresses excessive inflammatory responses. Conversely, age-associated dysbiosis contributes to a state of chronic low-grade inflammation, often described as inflammaging, which accelerates tissue degeneration and compromises skin resilience [166,167].

A key mechanistic link between the microbiome and skin aging lies in microbiome-derived peptides and bacterial molecular patterns. In this context, microbiome peptides include both peptides directly produced by skin commensals and host or synthetic peptides that modulate microbial ecology. Skin-resident bacteria synthesize a wide spectrum of antimicrobial peptides, which provide competitive advantages within microbial communities. Importantly, these peptides often exhibit selective activity, suppressing opportunistic or pathogenic species while sparing commensals [224]. Such selective pressure contributes to microbiome stability and indirectly protects the host.

Host defense peptides, in turn, are central regulators of microbial colonization. By shaping the microbial landscape rather than sterilizing it, these peptides support a dynamic equilibrium that is essential for long-term skin health. The bidirectional interaction between microbial peptides and host immunity mirrors broader principles of immune homeostasis regulation by bacterial components. Extensive studies on muramyl peptides, which are low-molecular-weight fragments of bacterial peptidoglycan, have demonstrated their capacity to act as potent immunomodulators rather than mere pathogen-associated signals [225,226].

Muramyl peptides interact with pattern recognition receptors, including NOD-like receptors, thereby modulating innate immune responses [227]. Their effects are dose-dependent and context-specific, allowing fine regulation of cytokine production, macrophage activation, and epithelial defense mechanisms. Importantly, these peptides do not induce an uncontrolled inflammation but instead promote immune balance, a property highly relevant to aging tissues prone to inflammatory dysregulation. Experimental and clinical studies have shown that muramyl peptide derivatives can correct immune deficiencies, normalize microbial landscapes, and enhance resistance to infections without compromising safety [228,229].

Structural studies of bacterial polysaccharides and peptidoglycan fragments, including O-specific polysaccharides of *Yersinia kristensenii*, have provided foundational insights into how defined bacterial structures interact with the immune system [230,231,232]. These works highlight that subtle variations in molecular architecture can dramatically influence biological activity. Such structure–function relationships are equally relevant for skin microbiome peptides, where minor chemical modifications may shift immunological outcomes from pro-inflammatory to regulatory [233,234,235].

Beyond innate immunity, microbiome peptides influence adaptive immune responses by shaping antigen presentation and T-cell polarization [236]. Epitope mapping studies using phage display have demonstrated how microbial and viral peptides interact with immune receptors, underscoring the precision with which peptide structures determine immune recognition [237]. Translating these principles to the skin suggests that microbiome-derived peptides may contribute to immune tolerance toward commensals while maintaining vigilance against pathogens, a balance that deteriorates with age.

Inflammation regulation by bacterial molecular patterns represents another critical axis linking microbiome peptides to skin senescence. Controlled activation of inflammatory pathways is essential for tissue repair and defense, whereas a chronic activation accelerates aging. Reviews on inflammation regulation by bacterial components emphasize that microbial signals can either exacerbate or suppress inflammation depending on molecular context and host condition [238]. In aged skin, where baseline inflammatory tone is elevated, regulatory microbial peptides may help restore balance and slow degenerative changes.

Emerging evidence also connects microbiome peptides with transcription factor regulation, affecting gene expression programs involved in oxidative stress responses, cell proliferation, and apoptosis. Pharmacological modulation of transcription factor activities is increasingly recognized as a strategy to counteract aging processes, including those in the skin [239]. Microbial peptides, acting upstream through immune and metabolic pathways, may indirectly influence these transcriptional networks [240].

The safety and bioavailability of microbiome-derived peptides are crucial for their therapeutic translation. Comprehensive analyses of bacteriocins and muramyl peptides (GMDP) indicate favorable safety profiles, minimal systemic toxicity, and potential for topical or localized application [241,242]. This makes them particularly attractive candidates for dermatological interventions aimed at aging skin, where long-term tolerability is essential.

Current data on microbiome-derived peptides allow us to consider them not simply as antimicrobial factors, but as low-molecular bioregulators influencing key mechanisms of inflammation and immune homeostasis in the skin.

### 5.1. Marine and Human Host Defense Peptides Influence on Microbiome Homeostasis of Skin

Symbiotic bacteria help maintain homeostasis of the skin by producing different peptides that inhibit pathogens. For example, *S. epidermidis*, a common commensal, produces phenol-soluble modulins and proteases that inhibit *S. aureus* biofilm formation, as well as AMPs, for example, epidermin [243]. *S. epidermidis* has been shown to defend against *S. aureus* invasion, and one of the mechanisms is realized through a secreted molecule that activates TLR2 in the skin, leading to increased AMP production by the host [244,245].

It has been revealed that *S. epidermidis* secreted the AMP epifadin, which displayed broad-spectrum activities but was a short-lived substance [246].

By introducing this strain to the skin microbiome, a factory for beneficial peptide production has been created, which keeps opportunistic pathogens at bay and reduces inflammatory signals. Clinical trials applying live *S. epidermidis* or *Roseomonas mucosa* strains to atopic dermatitis patients have shown improvement in disease severity, presumably because these microbes rebalanced the ecosystem and produced factors that healed the skin [247,248].

Postbiotics are soluble factors produced by probiotics, including peptides. Instead of live bacteria, one can apply these factors directly. For instance, a ferment lysate of *Vitreoscilla filiformis* culture is used in skincare. It likely contains bacterial metabolites and peptides that stimulate skin defenses and barrier function [249].

#### 5.1.1. Microbiome Editing with Phage-Peptide Conjugates

Bacteriophages can be engineered to deliver genes encoding AMPs or other peptides into target bacteria, causing them to self-destruct or modulate their behavior. This is a futuristic approach, but using a phage, one might introduce a gene into *Cutibacterium* acnes in the skin that makes it possible to produce an anti-inflammatory peptide, thus turning a once-inflammatory microbe into a soothing one [250].

#### 5.1.2. Enhancing Host-Microbe Signaling

Commensal microbes communicate with the skin’s immune system through pattern molecules and small metabolites [251]. Commensal-derived small molecules activate pathways in keratinocytes that lead to AMP production and anti-inflammatory effects [252].

For example, *S. epidermidis* strains produce a lipopeptide which transduces signals via TLR2/CD36 [93,253]. This results in the increase of production of human beta-defensins 2 and 3, fortifying the skin barrier against pathogens. These molecules are considered candidate therapeutic agents that might be applied to the skin in order to get the beneficial effect without using microbes per se. This approach might be especially helpful for older individuals whose microbiome naturally could not produce an adequate level of these signal molecules. By enhancing AMP production through commensal signals, it might be possible to compensate for aging-related declines in innate immunity.

#### 5.1.3. Microbiome Equilibrium Restoration

Aging is associated with reduced microbial diversity in the skin, and sometimes colonization by more virulent strains takes place. Microbiome transplants, based on taking a swab of microbes from a healthy young skin and applying it to older skin, are considered. This approach allows for essentially reintroducing a cadre of commensals that bring their peptide arsenals along. If those commensals take hold, they will continuously secrete AMPs and other beneficial peptides in situ. This “living” therapy might provide a long-term effect because microbes act as sustainable producers of peptides, maintaining homeostasis.

A healthy microbiome actively helps to keep the immune response balanced in the skin [93,254]. It has been shown that commensal bacteria could induce IL-10 production or regulatory T cells via their molecules, preventing excessive inflammation [255,256,257]. Some microbial peptides might be responsible for this immunoregulation [223,258,259]. Commensals producing such immunomodulatory peptides might reduce pro-inflammatory tendencies in the aged skin, thereby decreasing itch, reactivity, and chronic eczema that often discomforts elderly patients.

## 6. Structure–Activity Relationships and De Novo Design of Potent AMPs

**Figure 1:**
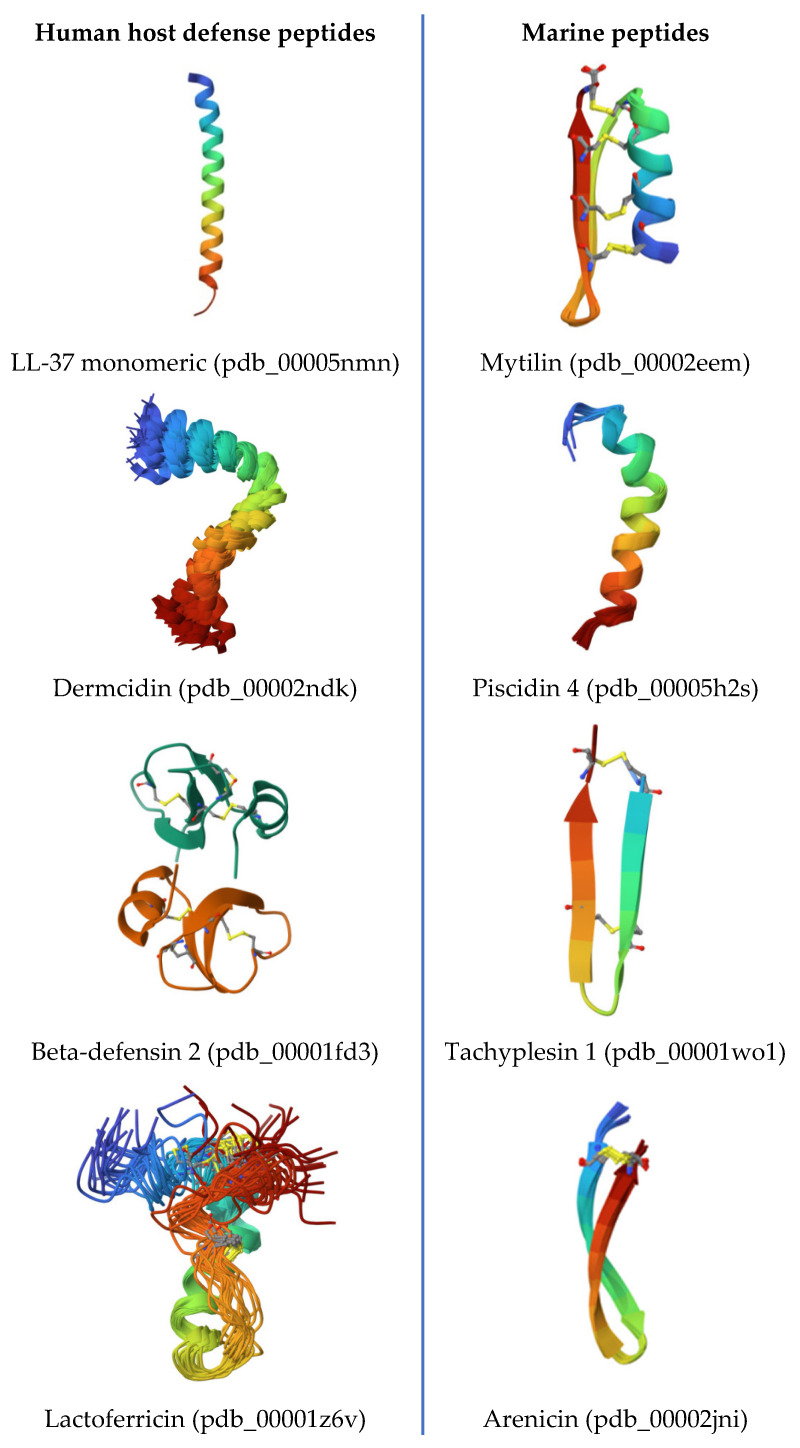
NMR structure of some AMP representatives. Human host defense peptides: LL-37 monomeric (pdb_00005nmn); dermcidin (pdb_00002ndk); beta-defensin 2 (pdb_00001fd3); lactoferricin (pdb_00001z6v). Marine peptides: mytilin (pdb_00002eem), piscidin 4 (pdb_00005h2s), tachyplesin 1 (pdb_00001wo1), arenicin 1 (pdb_00002jni).

Human and marine AMPs are the evolutionary earliest molecular factors of the innate immune system, playing a key role in host defense [260]. They have diverse structures classified in four main classes, including (i) α-helical, (ii) β-sheet, (iii) αβ, or (iv) (3) linear peptides, which are enriched with definite amino acid residues. Several well-known human and marine AMPs are presented in Figure 1.

Development of therapeutically valuable AMP can be based upon searching for novel AMP with the use of high-throughput biological or virtual screening, biophysical modeling, or design de novo by template-based approach [261]. AMPs with a potent antimicrobial activity can be used as template peptides for amino acid residue deletions or substitutions by site-directed mutagenesis. This approach allows to evaluate the contribution of specific amino acid residues to activity and toxicity manifestations.

Among the most active AMPs of human and marine animal origin are the peptides with a β-hairpin conformation, and marine β-hairpin AMPs stabilized by disulfide bridges belong to the most active and resistant to proteases molecules [111,262]. Analysis of the structure–activity relationship (SAR) of β-hairpin AMPs showed that amino acid substitutions had a significant influence on a spectrum of peptide activity and toxicity, but very seldom resulted in essential enhancement of antimicrobial activities [263,264]. In general, marine AMPs are amphipathic cationic molecules displaying bactericidal activities against a broad spectrum of pathogens and abilities to disrupt their membranes [265]. To create AMPs with a lower cytotoxicity and to better understand SAR in them, a number of potent recombinant analogs of marine peptides, such as tachyplesins [266,267], polyphemusins [262], and arenicins [172,173,268], were designed. In spite of the growing number of SAR investigations, activity and toxicity prediction are still not clear. Nevertheless, several structural and physicochemical characteristics, in particular hydrophobicity, amphipathicity, chain length, and cationicity, are among the most significant factors for AMP microbicidal and cytotoxic activities [269,270].

## 7. Signaling Pathways in Dermatology

Skin health is upheld by an integrated signaling network that links epithelial proliferation, differentiation, barrier function, pigmentation, wound repair, and innate immunity. Signaling pathways EGF and EGFR, IGF-1R, Hedgehog, WNT, MAPK, PI3K, MC1R-MITF, JAK-STAT are involved in growth, differentiation, and maintenance of stem cells [271]. Regenerative branch through the WNT signaling pathway also plays a critical role in skin development and mechanical-stretch-induced regeneration [272]. NF-κB, mTOR, TGF-β, AMPK-SIRT, complement, NLRP3, Notch, JAK-STAT, and MAPK signaling pathways organize skin inflammation [Ren]. It becomes obvious that dermatologic signaling is a cross-talking system, rather than isolated cascades.

At the tissue level, epidermal growth factor receptor (EGFR) and insulin-like growth factor 1 receptor (IGF-1R) function as major upstream growth and survival sensors. Their activation introduces a signal into Ras-Raf-MEK-ERK and PI3K-AKT-mTOR pathways, thereby regulating keratinocyte proliferation, migration, differentiation, and resistance to apoptosis. Hedgehog signaling, mediated through PTCH-SMO-GLI, is central to developmental patterning and tumor progression. WNT signaling controls stem-cell behavior and epithelial regeneration, whereas MC1R-MITF signaling governs melanocyte differentiation and melanogenesis. Cytokine-responsive JAK/STAT signaling translates inflammatory cues into transcriptional programs relevant to psoriasis, atopic dermatitis, alopecia areata, and wound inflammation [271].

Among the above-mentioned pathways, WNT signaling is especially important for a peptide-centered discussion because it links development, repair, and mechanotransduction. Bai et al. have described the canonical WNT/β-catenin pathway in which WNT binding to Frizzled/LRP5/6 inhibits the APC/Axin/GSK-3β destruction complex, allowing β-catenin to be accumulated and translocated to the nucleus, where it activates TCF/LEF-dependent transcription. In skin, this promotes keratinocyte migration and proliferation, hair follicle stem-cell activity, and fibroblast matrix production. In mechanically stretched skin, WNT signaling helps to convert physical force into regenerative growth. Moreover, ageing skin is not merely a tissue with reduced growth capacity; it is also a chronically inflamed tissue shaped by NF-κB, JAK-STAT, MAPK, NLRP3, mTOR, TGF-β, complement, Notch, and AMPK-SIRT balance. This point is central for host defense peptides, as many of them influence precisely on the same nodes [272,273].

AMPs realize their functions through cell surface receptors and downstream intracellular signaling pathways [274]. Human host defense peptides (HDPs) are among the examples of direct participation in cutaneous signaling. LL-37 expression is induced in wounded skin and promotes keratinocyte migration through HB-EGF-dependent transactivation of EGFR, followed by STAT3 activation [275]. This response is negatively constrained by SOCS1/Jak2-binding protein and SOCS3/CIS3, indicating that even a pro-repair peptide operates within a regulated signaling loop. Human β-defensins show related but distinct effects. hBD-2, hBD-3, and hBD-4 stimulate keratinocyte migration and proliferation, induce cytokine and chemokine production, mobilize intracellular Ca^2+^, and promote phosphorylation of EGFR, STAT1, and STAT3 [276]. In dermal fibroblasts, hBD-3 further accelerates wound repair through FGFR1/JAK2/STAT3 signaling, enhancing angiogenic growth factors, migration, and proliferation [277]. Taken together, these data indicate that endogenous HDPs act as local signaling mediators at the wound edge and function not only as direct antimicrobials.

The same peptides can also amplify disease-associated signaling when the tissue context changes. In psoriasis, LL-37 binds self-DNA and delivers it to endosomal TLR9 in plasmacytoid dendritic cells, thereby breaking tolerance and inducing type I interferon production [47,278,279]. LL-37 also complexes self-RNA and activates TLR7 in plasmacytoid dendritic cells and TLR8 in myeloid dendritic cells, leading to IFN-α, TNF-α, IL-6, and dendritic-cell maturation. In keratinocytes, LL-37 increases TLR9 expression and enhances type I IFN responses to CpG or genomic DNA [48,280]. In rosacea, LL-37 activates the NLRP3 inflammasome through lysosomal destabilization and promotes caspase-1/IL-1β-dependent inflammation [281]. Human β-defensins show a similar duality. hBD2 and hBD3 enhance the uptake of self or CpG DNA, stimulate TLR9-dependent IFN-α production in plasmacytoid dendritic cells, and, in psoriatic scales, cooperate with LL-37 to break innate tolerance to self-DNA [280]. hBD3 also promotes maturation and Th1-skewing activity of Langerhans cell-like dendritic cells. At the same time, hBD3 can be anti-inflammatory in atopic dermatitis-like disease, where it restores barrier function through aryl hydrocarbon receptor (AhR)-dependent autophagy [282]. Thus, human HDPs are best described as context-dependent amplifiers or restraints of inflammatory signaling rather than intrinsically pro- or anti-inflammatory molecules.

Microbiota-derived peptides and peptide-like bacterial products extend the same signaling logic. For classical bacteriocin-like molecules, the strongest mechanistic skin example is lugdunin from *Staphylococcus lugdunensis* [283]. Beyond direct antibacterial activity, lugdunin increases LL-37 and CXCL8/MIP-2 expression in keratinocytes and mouse skin, recruits monocytes and neutrophils through a TLR/MyD88-dependent mechanism, and synergizes with LL-37 and dermcidin-derived peptides against *Staphylococcus aureus*. Other commensal factors are not always classified as canonical bacteriocins, but they still act as microbiota-derived peptide signals. A sterile factor of <10 kDa from *S. epidermidis* activates TLR2 in keratinocytes, inducing hBD2 and hBD3 and strengthening antimicrobial defense [93]. Distinct *S. epidermidis*-secreted factors of <2 kDa activate AhR signaling, induce CYP1A1, IL-1α, IL-1β, and hBD3, and enhance epidermal defense in organotypic models [284]. Clinically, topical *Bacillus subtilis* bacteriocins reduced *S. aureus* abundance in acne lesions by 38% after 60 days in a pilot cohort, and in a 373-patient uncontrolled study reduced inflammatory lesions by 59%, non-inflammatory lesions by 58%, and GAGS scores by 56%. Although direct pathway mapping was not performed in that clinical study, the findings are consistent with microbiota-directed modulation upstream of TLR-, NF-κB-, and barrier-repair signaling [285].

Muramyl peptides deserve separate treatment because they are microbiota-derived peptidoglycan fragments with clear receptor binding and the specificity framework: NOD1 detects a diaminopimelate-containing muropeptide determinant of Gram-negative peptidoglycan, whereas NOD2 recognizes muramyl dipeptide (MDP), the minimal conserved motif common to all bacteria [227,286]. In epidermal cells, MDP-NOD2 signaling proceeds through RICK/RIP2 and TAK1 to activate NF-κB and MAPKs, thereby inducing cytokines and chemokines [287]. In keratinocytes, NOD2 also induces hBD-2, and this response requires NF-κB and AP-1 sites in the hBD-2 promoter [288]. This is a positive regulation of inflammation in the protective sense, as muramyl peptides strengthen epithelial alarm signaling and antimicrobial effector production. In vivo, NOD2 contributes to cutaneous defense against *S. aureus* by amplifying IL-1β-driven IL-6 responses that improve neutrophil killing [289]. An important mechanistic layer was added showing that NOD1-driven TNF production is strongly controlled not only at the transcription level but at the translational one through the p38-MNK-eIF4E axis [290]. NOD and TLR pathways often synergize to augment cytokine and antimicrobial peptide output [291]. This signaling is also relevant to downstream innate immunity, because innate-recognition pathways shape adaptive responses through dendritic-cell cytokine programs, and MDP can directly activate human NK cells through NOD2 and NF-κB while synergizing with IFN-α and IL-12 to increase IFN-γ production [292,293].

At the same time, muramyl peptides can negatively regulate inflammation. NOD2 has been shown to mediate anti-inflammatory signals downstream of TLR2 ligands, partly through altered IL-10 responses when NOD2 function is defective [294]. At the initial stage of exposure to muramyl peptides, inflammatory genes are activated; at later stages of exposure to them, transcription factor ATP3 and deubiquitinase A20, which control the inflammatory response, are activated [238]. More decisively, the chronic MDP exposure induces tolerance: in primary human macrophages, it suppresses subsequent TNF-α, IL-8, and IL-1β responses to NOD2, TLR2, and TLR4 restimulation, and this cross-tolerance is linked to reduced IRAK-1 signaling [295]. Secretory mediators deepen this brake, because IL-10, TGF-β, IL-1Ra, and mTOR-dependent circuits contribute to NOD2-mediated downregulation [296]. This provides a clear explanation for the apparent contradictory data: muramyl peptides are not simple inflammatory agonists but timing-dependent rheostats. Acute exposure can strengthen epithelial defense, whereas chronic exposure can impose homeostatic restraint. The obtained data support both sides of that model. Exogenous MDP delayed murine wound repair, reduced re-epithelialization, heightened inflammation, and increased β-defensin expression, indicating that excessive or mistimed NOD2 activation can impair tissue repair [297]. Positive regulation of inflammation by muramyl peptides occurs by sensing through NOD1/NOD2, which activates NF-κB, MAPKs, and, in some settings, p38-MNK-eIF4E-dependent cytokine translation. In skin and skin-relevant cells, this raises expression of cytokines, chemokines, antimicrobial peptides such as hBD-2, and IL-1β/IL-6-dependent antimicrobial defense. It also supports innate-to-adaptive crosstalk through dendritic-cell programming and NK-cell activation. Negative regulation occurs by chronic muramyl peptide exposure, which induces tolerance rather than escalation, including activating transcription factor ATP3 and deubiquitinase A20. NOD2 signaling can promote IL-10-associated anti-inflammatory bias, cross-tolerize macrophages to TLR2, TLR4, and IL-1R restimulation, and rely on IL-10, TGF-β, IL-1Ra, IRAK-1 downregulation, and mTOR-dependent mechanisms for this restraint. In skin repair, however, excessive exogenous MDP levels can be detrimental, delaying re-epithelialization and heightening inflammation. In summary, muramyl peptides are bidirectional regulators whose effect depends on dose, timing, cell type, and the presence of co-signals.

Marine AMPs largely converge on the same dermatologic signaling nodes as human peptides, which helps explain their therapeutic potential. Tilapia piscidin-derived peptides TP2-5 and TP2-6 stimulate HaCaT keratinocyte proliferation and migration through EGFR signaling, with downstream ERK, STAT3, and STAT5 phosphorylation [298]. They also enhance endothelial migration and neovascularization and upregulate collagen I, collagen III, and keratinocyte growth factor in fibroblasts. In mice, topical treatment has reduced the wound area from the second day and has accelerated closure. Epinecidin-1 similarly promotes HaCaT proliferation, vascularization, epithelial activity, and collagen deposition in MRSA-infected burn wounds, suppressing systemic CRP and IL-6 in a swine model and achieving complete healing within 25 days [123]. These studies suggest that effective marine peptides combine antimicrobial activity with activation of pro-repair EGFR-ERK-STAT and angiogenic programs while limiting excessive inflammatory signaling. Peptides from the marine single-celled golden-brown microalga *Isochrysis zhanjiangensis* have exhibited anti-photoaging potential via the MAPK/AP-1/MMP pathway and have displayed anti-apoptosis action [299].

Taken together, signaling pathways in dermatology are best viewed as a shared molecular framework in which barrier and immune cells, microbes, and peptides converge on EGFR/MAPK, PI3K-AKT-mTOR, WNT/β-catenin, JAK-STAT, AhR, NOD-TLR, and NLRP3 signaling pathways. Human HDPs, bacteriocins, muramyl peptides, and marine peptides can all support healthy skin, but only when they bias this network toward repair, controlled antimicrobial defense, and barrier restoration rather than toward self-nucleic-acid sensing or inflammasome overactivation. Therefore, the most attractive candidates for development of peptide-based therapeutics are those that preserve the pro-regenerative arms of EGFR, WNT, AhR, and JAK-STAT signaling while avoiding chronic LL-37-like amplification of TLR7/8/9 or NLRP3 pathways.

Thus, in skin, the pathways governing barrier maintenance, wound repair, pigmentation, and inflammation are deeply interconnected, and human, microbiota-derived, and marine host defense peptides act rather as context-dependent modulators than as simple antimicrobials.

## 8. Clinical Application of Human and Marine Host Defense Peptides in Dermatology

Clinical translation of host defense peptides (HDPs) in dermatology is heterogeneous. Among human HDPs, cathelicidin LL-37 has the most clinically relevant dataset. Convincing results have been obtained with LL-37 in chronic wound repair. In a first-in-man randomized placebo-controlled trial in venous leg ulcers, 34 participants received topical LL-37 twice weekly during a 4-week double-blind phase; the 0.5 mg/mL and 1.6 mg/mL doses produced approximately 6-fold and 3-fold higher healing rate constants than that with placebo, and mean ulcer areas fell by 68% and 50%, respectively, without safety concerns [300]. A later phase IIb multicenter trial in 148 patients did not show significant benefit in the full cohort, but post hoc analysis suggested improvement in the subgroup with large ulcers, defined as at least 10 cm^2^ at randomization [301]. A separate randomized double-blind diabetic foot ulcer trial revealed that baseline wound LL-37 levels were low in both groups, and the topical LL-37 trial increased the granulation index significantly on days 7, 14, 21, and 28, although it did not significantly reduce levels of IL-1α, TNF-α, or aerobic bacterial colonization [302]. Clinically, this pattern suggests that LL-37 may be more valuable as a pro-repair signal than as a simple topical anti-inflammatory or antimicrobial monotherapy. 

Defensin-based clinical trials are less pronounced and rather have anti-aging lines than disease-treating dermatologic ones. In a double-blind vehicle-controlled multicenter trial, 44 healthy women used a regimen containing α-defensin 5 and β-defensin 3 for 12 weeks; epidermal thickness increased significantly (*p* = 0.027), without histologic evidence of inflammation. Besides, visible pores, superficial wrinkles, oiliness, pigmentation, and skin evenness have been improved [303]. In a later open-label trial of 20 healthy patients, the same conceptual approach improved periocular wrinkling and elastosis over 90 days, with 30% rated “much improved” and 50% “improved” [304]. These studies have shown that defensin-based topical formulations might be tolerated and influence epidermal remodeling, but they are unlikely to be used as therapeutic agents in psoriasis, atopic dermatitis, acne, or infected wounds.

Microbiota-derived peptide strategies are clinically promising as they intervene at the level of ecological balance as well as host signaling. A live skin commensal, *Staphylococcus hominis* produces an arsenal of bacteriocins, including lantibiotics [305]. Topical administration of *Staphylococcus hominis A9* (*ShA9*) in a first-in-human, phase 1, double-blind, randomized 1-week trial in 54 adults with *S. aureus*-positive atopic dermatitis, met its primary safety endpoint, reduced *S. aureus,* and showed post hoc improvement in local eczema severity in the subgroup whose *S. aureus* strains were directly killed by *ShA9* [306]. In acne, a 1% cream containing *Bacillus subtilis* bacteriocins reduced *S. aureus* abundance by 38% in a 60-day pilot study of 12 patients; in an 8-week uncontrolled multicenter study of 373 patients, inflammatory lesions fell by 59%, non-inflammatory lesions by 58%, and GAGS scores by 56% [285]. These clinical studies are among the clearest signs that microbiota-derived peptide medications might be dermatologically useful. 

Muramyl peptides represent a different category of microbiota-derived c immunomodulators. Several drugs on the basis of muramyl peptides, including mifamurtide, lycopid, liasten, and polimuramil, have been advanced to the clinic [242]. The drug lycopid is sold in 9 countries without a prescription and is used to correct immunodeficiency conditions, including atopic disease, condylomatosis, soft-tissue infection, and furunculosis [242]. The strong direct effect was achieved in 86 patients with plaque psoriasis, and a significant improvement was reported in 50 patients (58.1%). An additional improvement in 9 patients (10.4%) was gained together with normalization of IL-4, IL-10, IL-12, TNF-α, sCD54, and MIF-related immune abnormalities [307]. Dermatologically, muramyl peptides are not “pro-inflammatory” or “anti-inflammatory” in any simplistic sense. Their clinical value consists rather in a potential capability to restore homeostatic innate signaling between flares.

A competitive marine environment has driven the evolution of defense peptides with unique structures and mechanisms of action [308,309].

Algae-derived peptides offer several advantages for dermatologic and cosmeceutical applications, including a high stability under saline conditions and a potent activity against multidrug-resistant pathogens [310]. Antioxidant peptide ETT from *Isochrysis zhanjiangensis* attenuates skin aging by maintaining homeostasis and promoting collagen generation [311]. The peptide protects skin cells (keratinocytes and fibroblasts) from oxidative stress caused by UVB radiation and hydrogen peroxide. It works by reducing reactive oxygen species (ROS) and activating the body’s natural antioxidant defense system. The peptides PYP1-5 and porphyra 334 from *Porphyra yezoensis f. coreana Ueda* increase the production of elastin and collagen [312].

A great number of investigations exhibited the efficiency of peptide extracts from seaweeds in skin repair, smoothing, and collagen generation [153,312,313,314,315,316,317,318,319,320]. Short peptides, including Lys-Val, Val-Arg, His-Ile, Lys-Leu, Ile-Leu, and Leu-Phe, Tyr-Phe, and Leu-Gly-Leu from *Spirulina platensis* and *Chlorella vulgaris,* significantly reduced activities of skin-aging enzymes, showing inhibition of elastase by 84%, collagenase by 90%, and tyrosinase by 66% [321]. A randomized clinical trial has shown that the peptides from *Spirulina platensis* in gel significantly improved wound healing, reduced inflammation, and alleviated post-surgical pain after periodontal flap surgery [322].

Thus, peptides from marine algae are highly effective natural compounds that are widely used in skincare to boost hydration, improve skin elasticity, and provide anti-aging benefits. They work by stimulating collagen production, retaining moisture, and inhibiting enzymes that cause skin sagging and breakdown. These peptides, along with other seaweed bioactive molecules, reduce inflammation, protect skin against UV damage, and support a youthful, radiant complexion.

Marine peptides are attractive mainly for their potential in wound healing and the treatment of infected burns. The tilapia piscidin 4 (TP4) stimulates fibroblast proliferation and increases collagen I, collagen III, and keratinocyte growth factor expression, while improving MRSA-infected wound repair in mice without obvious liver, kidney, or behavioral toxicity [298]. The related peptides, TP2-5 and TP2-6, enhance HaCaT keratinocyte proliferation and migration via EGFR signaling, increase fibroblast proliferation and extracellular matrix synthesis at low concentrations, promote angiogenesis, and accelerate murine wound closure. Epinecidin-1 derived from grouper *Epinephelus coioides* is even closer to translational relevance: in a swine MRSA-infected burn model, it promoted epithelial proliferation, vascularization, and complete healing within 25 days, whereas untreated burns did not heal over the same interval [123].

Overall, the medical application of human and marine HDPs in dermatology is in the phase of staged clinical translation so far. Their unique features and multifaceted functions make them promising candidates for developing new drugs for practical applications in dermatology.

## 9. Conclusions

Innovative strategies at the interface of microbiology and dermatology are directed at utilizing peptides to restore microbial equilibrium and skin homeostasis. Approaches such as introducing beneficial bacteria, applying their peptide products, or using targeted peptides to eliminate undesirable microbes aim to establish a positive feedback loop. A balanced microbiome secretes peptides and other signal molecules that maintain the skin barrier and immune stability, fostering an environment conducive to commensal survival. These interventions have the potential to improve skin health, reduce infections and inflammation, and to slow processes of skin aging and senescence by preserving a more youthful and resilient skin ecosystem.

Ultimately, the synergy among marine and human host defense peptides together with microbiome-derived peptides constitutes a comprehensive toolkit for supporting healthy skin. By drawing on strategies evolved in nature, both within the human body and in marine environments, scientists and clinicians are developing multifaceted interventions to maintain skin integrity, combat disease, and slow the progression of skin aging. Ongoing research and translational efforts suggest that future skin treatments will increasingly rely on biologically inspired therapies that engage the skin’s innate defenses and its microbial partners in promoting healing and protection.

## Data Availability

No new data were created or analyzed in this study. Data sharing is not applicable to this article.
